# Crosstalk with infant-derived Th17 cells, as well as exposure to IL-22 promotes maturation of intestinal epithelial cells in an enteroid model

**DOI:** 10.3389/fimmu.2025.1582688

**Published:** 2025-05-01

**Authors:** Zohreh Sharafian, Paula T. Littlejohn, Christina Michalski, James A. Sousa, Janelle Cheung, Mariana Hill, Hannah Piper, Kevan Jacobson, Pascal M. Lavoie, Joannie M. Allaire, Bruce A. Vallance

**Affiliations:** ^1^ Department of Pediatrics, University of British Columbia, Vancouver, BC, Canada; ^2^ British Columbia Children’s Hospital Research Institute, Vancouver, BC, Canada; ^3^ Department of Medical Genetics, Centre for Molecular Medicine and Therapeutics, Vancouver, BC, Canada

**Keywords:** IL22, Th17, enteroid models, infant, neonate, RNAseq, intestinal development, organoid

## Abstract

**Introduction:**

The intestinal epithelium of human infants is developmentally immature compared to that of adults. Exactly how this immaturity affects key epithelial functions and their interactions with nearby immune cells remains an understudied area of research, partly due to limited access to non-diseased infant gut tissues. Human intestinal organoids, or “mini guts” generated from tissue stem cells, are promising models for investigating intestinal biology and disease mechanisms. These three-dimensional structures closely mimic their tissue of origin, including cellular physiology and genetics. We have also previously shown that neonatal Th17 cells represent a distinct cell population with a cytokine profile skewed toward IL-22 production rather than IL-17A, as seen in adult Th17 cells.

**Methods:**

In this study, we sought to model the impact of neonatal-derived Th17 cytokine, namely IL-22 and the intestinal epithelium using infant-derived ileal enteroids. We generated enteroids from ileal biopsies from infants (< 6 months old) and cultured them for seven days with standard organoid growth media, organoid media supplemented with conditioned media from cord-blood-derived Th17 cells, or media supplemented with recombinant IL-22. We assessed morphological changes and conducted transcriptomics profiling via RNAseq.

**Results:**

Exposing enteroids to neonatal Th17-cells-derived conditioned media led to enhanced growth, maturation, and differentiation as compared to control media. These effects were ablated when an IL-22 neutralizing antibody was used, while conversely, supplementing with recombinant IL-22 mimicked the Th17 effects, increasing intestinal epithelial cell proliferation and inducing marked differentiation of secretory cells. Our transcriptomic profiling similarly demonstrated significant changes in response to IL-22 with downregulation of Wnt and Notch signaling and upregulation of immune pathways, particularly interferon signaling. The transcriptomic data also suggested that IL-22 treatment led to changes in cell type composition with an increase in stem- and progenitor cells at the expense of enterocytes.

**Conclusion:**

Taken together, our data suggests that early-life intestinal development is likely influenced by IL-22-dependent crosstalk between the infant epithelium and exposure to neighboring Th17 cells. This promotes epithelial cell maturation and immune readiness, reflected at both the morphological and molecular levels. Our work also provides a relevant framework for studying healthy infant gut development, which can be further leveraged to examine early-life gastrointestinal disorders, model complex human disease, and therapeutic testing while reducing reliance on animal models.

## Background

The intestinal epithelium comprises a single continuous layer of columnar intestinal epithelial cells (IECs) that line the entire gastrointestinal (GI) tract (1, 2). Infant IECs are both structurally and developmentally immature at birth, but following the infant’s initial colonization by gut microbes and their first ingestion of food, these cells undergo a complex and highly regulated maturation process that extends into childhood (1, 2). During this period, antigenic stimulation from both microbial and dietary sources, as well as the release of growth factors and other molecules from underlying immune and stromal cells, drives the functional development of the gut (2, 3). This involves the deepening of intestinal crypts and lengthening of villi to protect the stem cells that localize to the crypt bases. Moreover, intestinal stem cells can also differentiate into specialized subtypes, such as Paneth cells which produce antimicrobial factors, and goblet cells, which produce glycosylated mucins that form the overlying and protective mucus layer (2, 4).

Functionally, the IECs and the mucus layer together form a critical intestinal barrier, separating the underlying mucosal immune system from the microbes and food antigens found in the lumen. This barrier limits the passage of antigens from the lumen and into the mucosa, where they can trigger inflammation, as seen in conditions such as necrotizing enterocolitis (NEC), a severe pathology that develops in a subset of prematurely born infants (5). Both animal and human studies have shown significant morphological and functional differences between the infant and adult gut (6). For example, before birth, intestinal goblet cells are relatively immature and express only modest levels of secreted mucins such as MUC2, MUC3, and MUC5b, leading to a relatively thin GI mucus layer (4). As the infant and their goblet cells mature, mucin production increases and the mucus layer significantly thickens, but until then, the immature mucus layer leaves the intestinal mucosal surface vulnerable, especially in premature neonates, significantly increasing their risk of NEC or sepsis following the translocation of pathological bacteria through the gut mucosa (2, 7–9).

Globally, infectious and non-infectious GI disorders among children are rapidly increasing (10–12), but we lack mechanistic insights into the natural development of the gut. This underscores an urgent need to develop models to expand our capacity to investigate gut maturation and to examine the GI tract in health and disease states. Such advances could also be key to devising therapeutic strategies in the era of personalized medicine (13). Nevertheless, despite their relatively immature GI tract, infants are not entirely devoid of age-adapted gut protective mechanisms. Indeed, emerging evidence suggests that infants heavily depend on the actions of their IECs for protection, which may employ sophisticated mechanisms to protect mucosal tissues from environmental insults until fully matured (14–16). For example, tightening of epithelial barrier junctions and increased lysozyme production by Paneth cells have been observed in infants shortly after birth, processes presumably aimed at preventing the passage of microbes and their products across the GI epithelium (14). Goblet cells, which are high in numbers in neonates and infants, contribute to immune responses and education through the delivery of antigens via the so-called goblet cell-associated antigen passages (2, 7). Nonetheless, data on the developmental stages of gut maturation in humans are lacking, in large part because of the difficulty in accessing sufficient gut tissue material for experimental studies.

Clearly, studying the GI tracts of human infants poses significant ethical and practical challenges, highlighting the necessity for establishing *in vitro* gut models that can accurately represent early-life gut physiology. Organoids are three-dimensional (3D) cell clusters derived from tissue-specific, induced pluripotent, or other stem cells that are cultured in nutrient-rich media enriched with stem cell niche factors, such as Wnt, Noggin, and R-spondin-1 (WRN), as well as growth factors, and an *in vitro* gel-based extracellular matrix or in suspended hydrogel (17–20). This environment stimulates signaling pathways that promote the cellular differentiation and growth of the intestinal stem cells (17–19), which recapitulates the structure and function of the tissue of origin with great fidelity. For example, stem cells isolated from the small intestine generate “enteroids” that mimic the small intestinal epithelium, whereas colonoids are derived from stem cells located in the large intestine, and they model the colonic epithelium. Furthermore, when properly stimulated and cultured, organoids can differentiate into diverse cell subtypes, enhancing their complexity, accuracy, and usefulness in understanding normal development and disease pathology. In this work, we focused on organoids derived from stem cells isolated from infant ileal biopsies.

Notably, under standard organoid culture conditions, intestinal organoids lack the necessary environmental signals that help shape the function of the gut epithelium *in vivo* (21). For example, we recently showed that mouse cecal organoids require stimulation with the cytokine interferon-gamma to respond to enteric pathogen infections in a manner mimicking that seen *in vivo* (22). Similarly, in prior studies using adult colon biopsies from healthy controls, we (23) found that culturing colonoids with the supernatant from type 1 regulatory T-cells (Tr1) promoted goblet cell differentiation. Despite the supernatants containing high levels of the cytokines interleukin (IL)-10 and IL-22, the addition of neutralizing antibodies against these cytokines did not inhibit the goblet cell phenotype, indicating that in this adult organoid model, goblet cell differentiation was independent of the actions of IL-10 and IL-22 (23). This, however, contrasts with other studies showing that IL-22 enhances transit amplifying (TA) and secretory cell differentiation (24–26).

While numerous studies have explored the development of the infant gut microbiome during the first year of life, there is a notable scarcity of research explicitly examining the human gut mucosa within this critical period (27, 28). To address this gap and to define the baseline functions in infant intestinal epithelium and the potential regulatory role of neonatal Th17 cell-derived cytokines, and particularly IL-22, we developed an enteroid model derived from ileal biopsies collected from infants less than 6 months of age. We showed that co-culturing these enteroids with neonatal Th17 T-cell conditioned media stimulated proliferation, mucus secretion, and antimicrobial peptide production in an IL-22-dependent manner (23, 29). Furthermore, RNA-sequencing-based genome-wide transcriptome profiling of these infants’ enteroids showed that IL-22 significantly shaped the transcriptome with regarding to cellular identity, maturation, immune/inflammatory response, and barrier function.

## Methods

### Ethics statement

The study was approved by the University of British Columbia’s Children’s & Women’s Research Ethics Board (#H19-02989 for intestinal biopsies and #H14-03199 for cord and adult peripheral blood). Parental written informed consent was obtained for the collection and use of all specimens.

### Generation, culturing and maintenance of infant -derived enteroids

We generated enteroids from ileal biopsies collected from male and female infants <6 months of age undergoing a scheduled intestinal diagnostic procedure at the BC Children’s Hospital. Only samples from non-inflamed tissues were used to generate enteroids in this study. The de-identified specimens were then transferred to the research team at BC Children’s Hospital Research Institute. Using previously established methods (21) tissue samples were washed and resuspended in a cold chelating solution (CCS) with 0.5 M EDTA and 100 µL of Gentamicin. Samples were incubated at 4°C on a shaker (600 strokes/minute) for 45 mins. Tissues were then finely chopped under sterile conditions and resuspended in cold MEM+++ (advanced DMED/F12 supplemented with 1% 1X Penicillin/Streptomycin, 1% 1X Glutamax, and 1% 1M HEPES). After washing, the supernatant was removed, and cold MEM+++ and Matrigel (Corning) was mixed with the sample. Matrigel domes of 30 µL were placed in the center of each well in a pre-warmed 24-well culture plate and incubated at 37°C (5% CO_2_) for 15–30 mins to ensure solidification of the Matrigel. Pre-warmed enteroid growth medium (500 µL) was added to each well and incubated at 37°C.

Every two days, 400 µL of enteroid growth media was removed and replaced with an equal amount of fresh media (advanced DMEM/F12, supplemented with 1% 1X Pencillin/Streptomycin, 1% 1X Glutamax, and 1% 1M HEPES) and 50% WRN supplemented with N2 (Invitrogen), B27 (Invitrogen), N-acetylcysteine (Sigma-Aldrich), nicotinamide (Sigma-Aldrich), EGF (Invitrogen), A83-01 (Tocris), SB 202,190 (Sigma-Aldrich) and Y-27632 (AbMole). Enteroids were passaged every 7 to 10 days until deemed appropriate for further experimentation. Enteroids were used only between passages 3 and 5. To passage the enteroids, Matrigel domes were disrupted with cold PBS including 5 mM EDTA and incubated at 4°C for 30 mins. Enteroids were then washed twice with cold media and broken with vigorous pipetting (50x, 3 rounds) and vortexing. Disrupted enteroids were centrifuged (211 RCF/1500 rpm at 4°C for 5 mins), the supernatant removed, then resuspended in the proper volume of Matrigel.

### Naïve CD4 T cell isolation and culture

Cord blood was collected from term Cesarean section deliveries (>38–41 weeks of gestation) in sodium heparin anti-coagulated vacutainers (Becton Dickinson, Canada) and processed within 2–6 hours of collection. Cord blood mononuclear cells (CBMCs) were isolated from de-identified cord blood samples via density centrifugation with LymphoPrep (STEMCELL Technologies), cryopreserved in PBS with 44 mg/mL human serum albumin, 2 mM EDTA, and 10% DMSO (1.0 x 10^6^ cells/mL), and stored at -80°C until further processing. Naïve CD4+ T cells were isolated from thawed CBMCs via immunomagnetic negative selection (StemCell Kit) manually counted and seeded in a 96-well round-bottom plate (0.5 x 10^6^ cells per well). In this study, we used three pooled batches of cord blood samples. Each batch consisted of four cord blood samples from individual patients, where naïve T cells were differentiated into Th17 cells. The supernatant (i.e., conditioned media) from each batch of cord-blood-derived Th17 cells was collected. Pooling batches helped minimize biases. As previously described (29, 30), activation of CD4 T cells was achieved using anti-CD3 and anti-CD28 soluble antibody complexes (25 µL/mL; ImmunoCult™ Human CD3/CD28 T Cell Activator) in complete Roswell Park Memorial Institute Medium, cRPMI (RPMI with 10% human AB serum, 1% sodium pyruvate, 1% 1X Penicillin/Streptomycin) in the presence of human recombinant IL-6 (20 ng/mL), IL-23 (10 ng/mL), IL-1β (10 ng/mL) (Peprotech) and neutralizing IL-4 and IFN-γ antibodies (BD Pharmingen) at 37°C (5% CO_2_) for seven days, with fresh media and cytokines added after 3 days. Conditioned media (i.e., from neonatal Th17 cells) was collected at the end of the culture and frozen at -80°C in batches. We further determined the IL-22 concentration in culture supernatants by ELISA (Biolegend IL-22 Kit) to be 100ng/ml. (**Supplementary Protocol**) This concentration was then used to carry out further analysis.

### Stimulation of infant enteroid cultures

Enteroids were first grown in the organoid growth medium (advanced DMEM/F12 used as base media, supplemented with 1% 1X Penicillin/Streptomycin, 1% 1X Glutamax, and 1% 1M HEPES) and 50% WRN supplemented with N2 (Invitrogen), B27 (Invitrogen), N-ace-tylcystine (Sigma-Aldrich), nicotinamide (Sigma-Aldrich), mEGF (Invitrogen), A83-01 (Tocris), SB 202,190 (Sigma- Aldrich), Y-27632 (AbMole) for 3 days (400 μL/well). Following this, organoid media was replaced with fresh 70% organoid growth medium supplemented at 30% either with neonatal Th17-conditioned media, rh IL-22 (100 ng/ml), cRPMI as a control condition, or neutralizing IL-22 (R&D Systems) at 5 μg/ml. Th17 condition was incubated with neutralizing IL-22 for 3 hours at 37 degrees and then added to the enteroids. Culture conditions were refreshed in organoid media every 2 days. On Day 7, organoid cultures were collected, pooled, and harvested for further analysis.

### Harvesting and fixation of human enteroids for immunofluorescence staining

Enteroids were harvested as previously described (31). In brief, Matrigel domes were disrupted using cold 5 mM EDTA in PBS, spun, and washed in PBS. Harvested enteroids were fixed with 4% paraformaldehyde, resuspended in melted 3% agarose with PBS, solidified, and placed in 70% ethanol until further histological processing. Fluorescence intensity of Ki67 and MUC2 in stimulated infant enteroids were semi-quantified using Fiji ImageJ’s integrated density tool. Values were normalized to total DAPI per enteroid. All samples within the experiment were stained under the same conditions.

### Brightfield imaging of organoids

Organoids were imaged using a Brightfield microscope (Olympus Corporation, Tokyo, Japan) at 4x magnification. For imaging, the culture plate was placed directly on the microscope stage, and images were acquired using (Cellsens Entry, version 4.3) with consistent exposure settings across all samples. The budding number and organoid area for each organoid were measured using Fiji ImageJ software. 5–10 organoids and 30 organoids per condition were randomly selected for the measurements of buds and surface area, respectively.

### Immunofluorescence and staining of enteroid cultures

As previously described (21), immunofluorescence staining was conducted using the following primary antibodies, with dilutions as follows: Ki67 (rabbit, 1:100, 3MA5-14520, Invitrogen), mucin 2 (rabbit, 1:200, A01212, Boster), epithelial cadherin (mouse, 1:100, 610182, BD transduction laboratories). Secondary antibodies included Alexa Fluor 488 or 568 (1:2000). ProLong Gold antifade reagent containing 40,6-diamidino-2-phenylindole (DAPI, Invitrogen) was used to mount slides. Antigen retrieval was performed using freshly prepared citric acid buffer (pH 6.0). A Zeiss Axiomager Z1 (Carl Zeiss, Oberkochen, Germany) at 20X magnification and an AxioCam HRm camera with AxioVision software (version 3.0) were used to view and image slides.

### RNA extraction and profiling of enteroid cultures

Following previously described protocols to collect RNA (31), pooled enteroids from three to four wells treated with control media, or media supplemented with rhIL-22 (100ng/ml), were resuspended in 300-600 μl TRIzol (Invitrogen) and lysed at room temperature for 15 minutes. RNA purification was performed with the Direct-zol RNA Microprep Kit (Zymo Research) following the manufacturer’s protocol. A NanoDrop ND-2000 spectrophotometer (Thermo Fisher Scientific) was used for the quantification of isolated RNA.

### RNA sequencing analyses

Pooled enteroids under control and rhIL-22 treated conditions were subjected to RNAseq profiling. Sample quality control was performed using the Agilent 2100 Bioanalyzer. Qualifying samples were then prepped following the standard protocol for the NEBnext Ultra ii Stranded mRNA (New England Biolabs). Sequencing was performed on the Illumina NextSeq2000 with Paired-End 59bp × 59bp reads. Sequencing data were de-multiplexed using Illumina’s bcl2fastq2. De-multiplexed read sequences were then aligned to the Homo sapiens/Mus Musculus (PAR-masked)/hg19 or mm10) reference sequence using STAR aligner (33). Assembly and differential expression were estimated using Cufflinks (http://cole-trapnell-lab.github.io/cufflinks/) through bioinformatics apps available on Illumina Sequence Hub.

RNAseq data analysis and heatmaps were made using R version 4.3.1 and R studio version 2023.06.2. Some of the figures were generated in Prism 10.2.3. Samples were batch-corrected using ComBat_Seq. A paired control-treatment model was fitted for differential gene analysis using the standard edgeR workflow. Gene set enrichment analysis (GSEA) was performed on a ranked gene list with -log10 (P-value) *sign (log fold change) as the input metric. Gene sets were obtained from curated databases (KEGG, Hallmark, GO), and 1000 permutations were performed to calculate enrichment. Sample level enrichment analysis (SLEA) was performed as described previously (32) using marker genes previously obtained by single-cell RNA-seq analysis of the human intestine (33). Multiple testing correction was done using the Benjamini-Hochberg method, and a false discovery rate of 5% was considered significant.

### Statistical analysis

Data are representative of at least 3 biological replicates in independent experiments, except where otherwise noted. The results are expressed as the mean values ± standard errors (SEM). Mann-Whitney U-test, t-test, and two-way ANOVA were assessed using GraphPad Prism software, version 9.4. P-value of ≤ 0.05 was considered significant, with asterisks denoting significance in the figures.

### Data availability

Gene expression data have been submitted to the NCBI Gene Expression Omnibus repository under the accession number GSE282290.

## Results

### Neonatal Th17 cell-derived conditioned media supports the growth of infant enteroids

**Figure 1:**
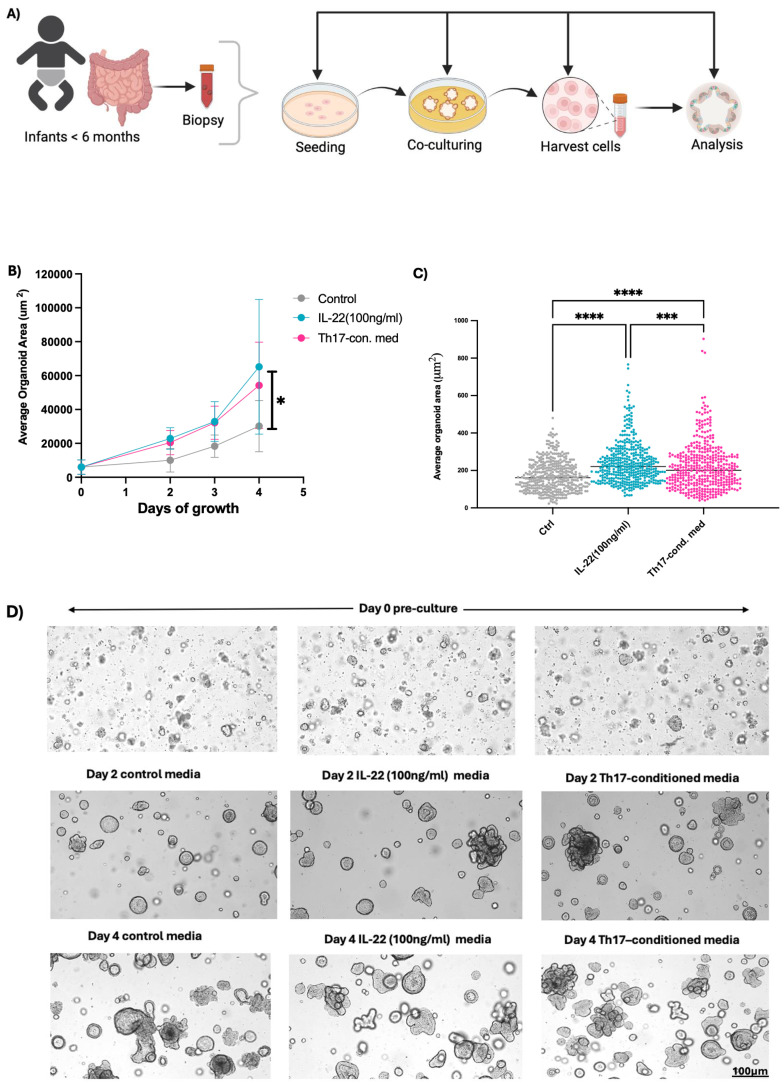
Neonatal Th17 cell-derived cytokines support infant enteroid growth. **(A)** Enteroids generated from biopsies (n=3) collected from infants < 6 months old were cultured in standard organoid media, or with either Th17-conditioned media or media supplemented with rhIL-22 and subjected to downstream analysis. **(B, C)** Infant enteroids cultured in Th17 -conditioned media or with rhIL-22 for 4 days experienced differential growth phenotype, in terms of size (average organoid area um^2^/days of growth) as compared to control conditions, with the Th17-conditioned media having significantly larger size enteroids than controls. **(D)** Representative brightfield images of infant enteroids at Days 0, 2 and 4. Enteroids exposed to Th17-conditioned media and rhIL-22 showed more budding after 4 days of culturing (Scale bar, 100μm). Images 4x magnification. Technical replicates n=30; N= 3 individuals. Statistical significance in **(B)** was calculated using two-way ANOVA. *P ≤ 0.05, *** P <0.001, **** P <0.0001. Enteroid samples used N001, N002 and N005.

Our previous work showed that cord blood Th17 cells produced abundant levels of IL-10, IL-13, and were also notably skewed towards IL-22 production, away from producing IL-17A, compared to adults (29). To obtain insight into the natural development of infant IECs and to model their interactions with Th17 cells in the infant GI tract, we generated enteroids using biopsies obtained from infants undergoing scheduled diagnostic procedures. Additionally, we generated Th17 cells from neonatal cord blood, as previously described (34). Similar to our previously published protocol (23, 29), we cultured infant enteroids in control enteroid growth media or in the same media supplemented with 30% media from stimulated, neonatal-derived Th17 cells (**Figure 1A**). Enteroids exposed to the Th17 conditioned media grew larger in terms of average enteroid area (μm^2^), than those grown in control media (**Figure 1B, Supplementary Protocol**). We saw a clear separation in growth rates between the two enteroid groups that reached significance by day 4. We next examined whether IL-22 mediated the phenotype displayed by enteroids exposed to neonatal Th17 condition media by culturing enteroids in control media supplemented with human recombinant IL-22 (100 ng/ml). Similar to the enteroids exposed to the Th17 condition media, the IL-22 exposed enteroids exhibited faster growth, nearly to the elevated level observed in the Th17-conditioned media exposed enteroids (**Figures 1B–D**). Moreover, the enteroids exposed to Th17-conditioned media and rhIL-22 displayed crypt-like structures with budding features which were observed as early as Day 2 of exposure, suggestive of greater cellular heterogeneity and increased structural mimicry of the *in vivo* gut (**Figure 1D**). In contrast enteroids cultured in the control media displayed more cystic enteroid phenotypes.

### Infant Th17-conditioned media increases enteroid proliferation and differentiation in an IL-22-dependent manner

**Figure 2:**
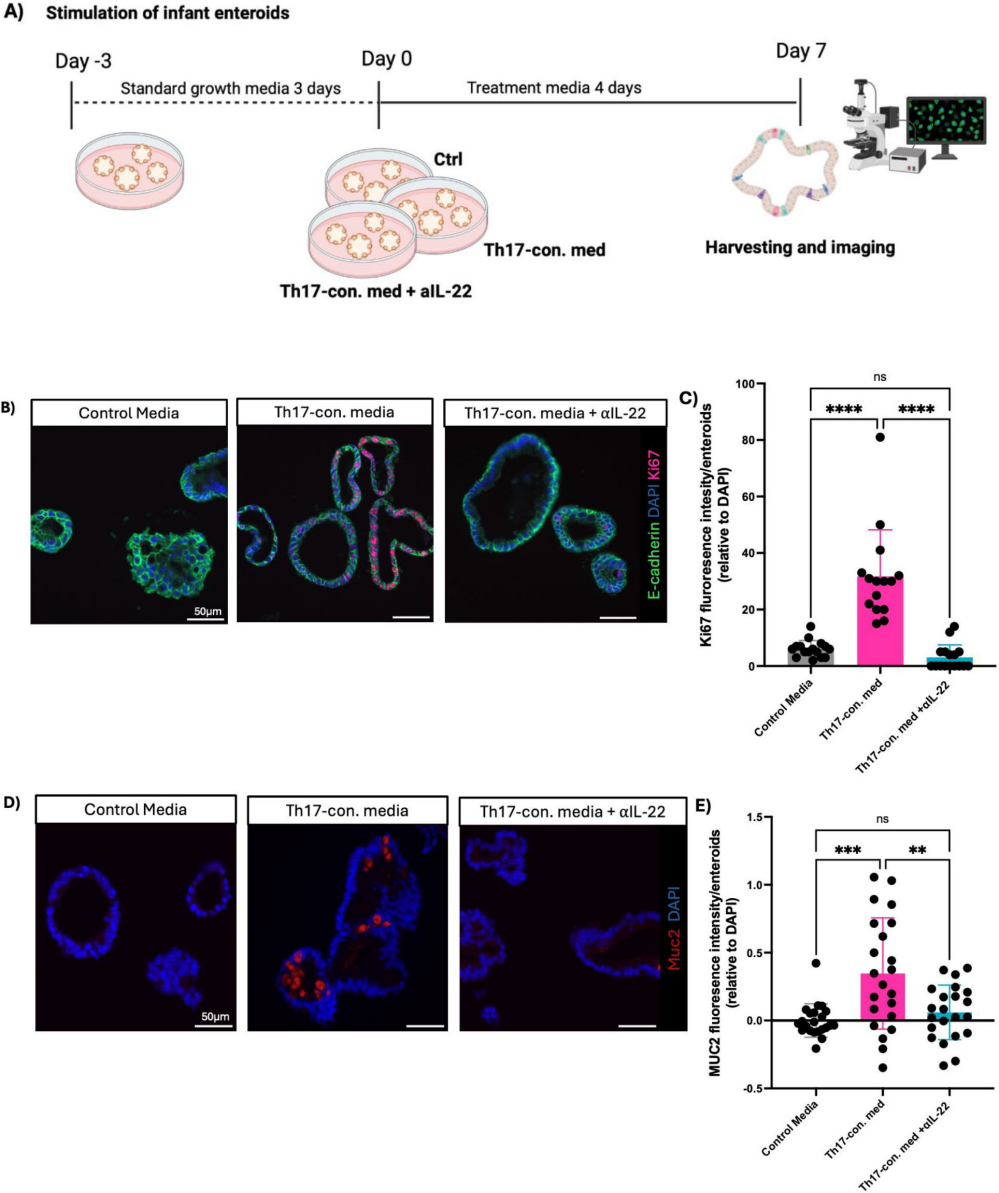
IL-22 is the key cytokine underlying the effects of the human infant Th17-conditioned media. **(A)** Experimental design for enteroid stimulation. **(B)** Representative immunofluorescent staining of Ki67 (red), E-cadherin (green), and DAPI (blue) in infant enteroids cultured in control media (left), control media supplemented with infant Th17-conditioned media (middle), or control media plus Th17-conditioned media plus neutralizing anti-IL-22 (right) (Scale bars, 50 μm). **(C, E)** Fluorescence intensity of Ki67 and MUC2 per enteroid relative to DAPI following exposure to Th17-conditioned media. (technical replicates n=5; N= 4 individuals, statistical difference calculated by one-way ANOVA). Enteroids exposed to infant Th17-conditioned media showed more goblet cell (MUC2) staining as compared to control, and Th17-conditioned media plus neutralizing IL-22 groups (Scale bar, 50 μm). **(D)** Infant enteroids stimulated by neonatal Th17-conditioned media display increased goblet cell numbers/size as indicated by increased MUC2 intensity per enteroid. MUC2 intensity was normalized to DAPI (technical replicates n=5; N= 4 individuals, statistical differences calculated by t-test **P < 0.01, *** P <0.001, **** P <0.0001) (Scale bar, 50 μm). Enteroid samples used N001, N002, N011 and N012.

Mucosal barrier integrity, epithelial repair, renewal, and overall gut homeostasis, especially during early development, depends on the rapid generation of new IECs from intestinal stem cells (ISCs) (2, 35). Thus, we explored whether increased cell proliferation was responsible for the subsequent larger enteroid sizes seen following exposure to the Th17-conditioned media in our model. We measured cellular proliferation by staining for Ki67, observing significantly higher Ki67 fluorescence intensity in the Th17-conditioned media infant enteroids as compared to the control group (**Figures 2A, B**).

In addition to cellular proliferation, differentiation into specialized cell lineages is also a key step in the maturation of the intestinal epithelium. Notably, secretory IECs, such as mucus producing goblet cells, are essential to the innate defense and barrier functions of the GI tract, thereby helping to safeguard the immature intestine from noxious luminal stimuli (2). We observed increased differentiation of goblet cells in those infant-derived enteroids treated with Th17-conditioned media, as compared to controls, as marked by a higher intensity of MUC2 staining (**Figures 2C, D**). We observed an ablated phenotype with no detectable MUC2 staining in enteroids treated with neutralizing αIL-22. These findings indicate that IL-22 is necessary to drive the increased cellular proliferation and differentiation phenotype seen in Th17-conditioned media stimulated infant enteroids.

### IL-22 induces differential gene expression linked to cellular identity and maturation

**Figure 3:**
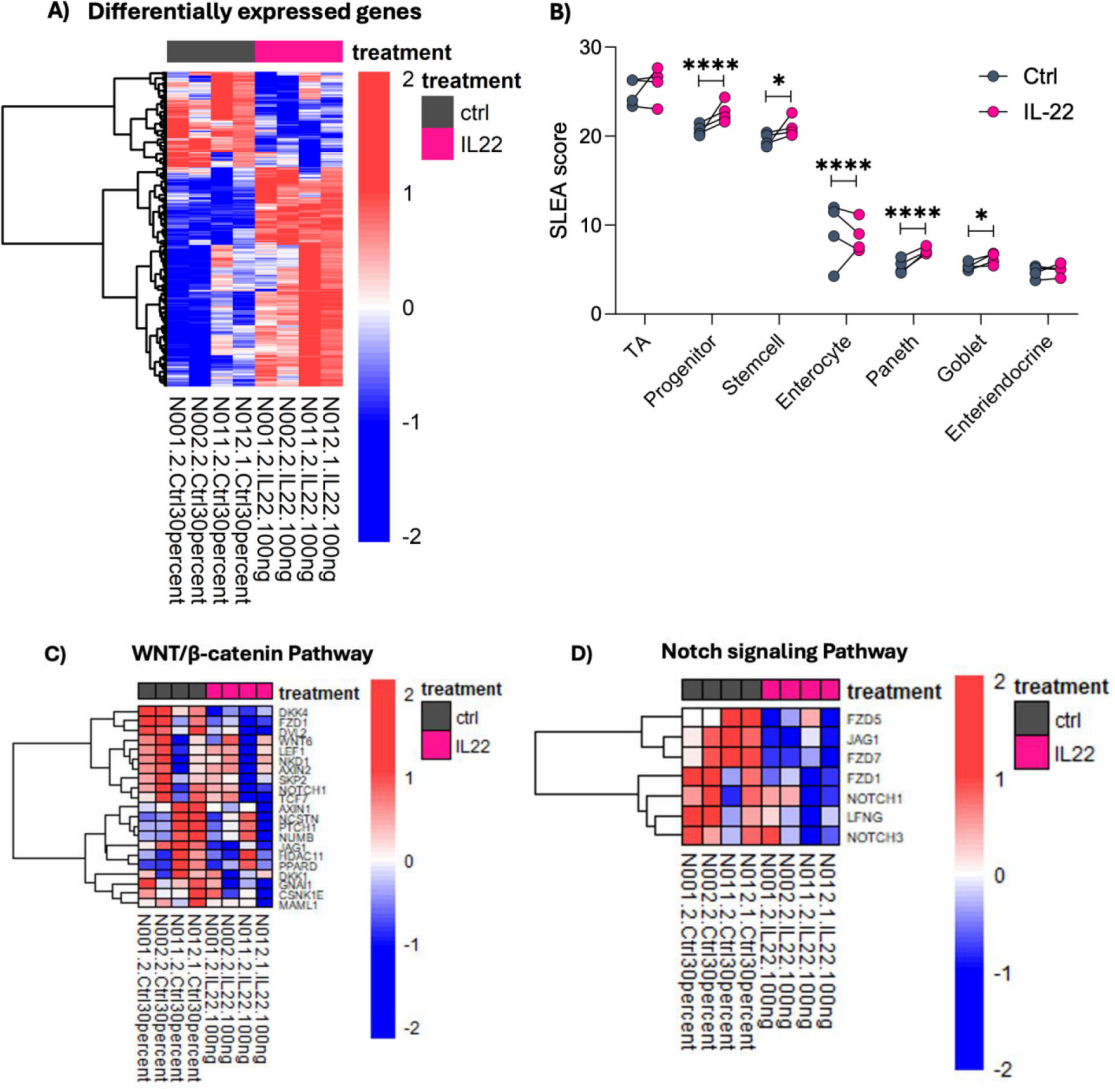
IL-22 induces differential gene expression related to cellular identity and maturation. **(A)** Heatmap depicts the Z score normalized expression of genes altered by IL-22 treatment (FDR < 0.05). **(B)** Enrichment for IEC subtypes following IL-22 treatment was calculated using GSEA based on marker genes obtained from Wang et al. (33). Depicted are the corresponding sample level enrichment analysis (SLEA) scores for each sample with asterisks indicating the FDR value from the GSEA. (*FDR < 0.05, ****FDR < 0.0001). **(C, D)** IL-22 favors IEC differentiation indicated by the downregulation of genes in WNT β-catenin and NOTCH signaling pathways. Depicted are the leading-edge genes from GSEA using Hallmark pathways. The heatmap color scale represents Z scores, with red and blue respectively showing increase and decreased expression. Enteroid samples used N001, N002, N011 and N012.

As the effects of Th17-conditioned media on infant enteroids were largely mimicked by rhIL-22, we further investigated how IL-22 impacts the cellular fate and function of infant IECs in promoting maturation and development. We performed RNA-sequencing on ileal-derived infant enteroids (N001, N002, N011 and N012, see sample table in Supplemental Material) that were cultured for three days in control media, followed by four days of culture with either rhIL-22 or control media (for a total of seven days). We found 251 differentially expressed genes in response to IL-22 treatment at FDR <0.05 (**Figure 3A**). We used previously published markers derived from single-cell RNA-seq analysis of the human intestine (33) to estimate proportions of cell subsets using gene set enrichment analysis (GSEA). Strikingly, IL-22 treatment led to a significant increase in signatures for Paneth, transient-amplifying (TA), progenitor, and stem cells and a reduced signature of enterocytes (**Figure 3B**; **Supplementary Figures 1A–G**). We note that although TA cells serve as progenitors for both absorptive and secretory IECs, and while much remains to be understood about their role in maintaining the stem cell niche (36), recent findings indicate that IL-22 drives their expansion (25), which is in line with our data. Overall, our results suggest that IL-22 induces changes in the overall cellular makeup of the enteroids.

Appropriate signaling between ISCs and IECs harmonizes the delicate balance required for epithelial cell growth and maintenance. Notch signaling is a highly conserved pathway important for driving IEC differentiation into absorptive (i.e., enterocytes) rather than secretory (i.e., goblet and Paneth) cell lineages, while Wnt signaling is important for epithelial renewal and ISCs maintenance (35, 37). Data from our transcriptomic profiling showed a marked downregulation of both Wnt/β-catenin and Notch signaling pathways in response to IL-22 (**Figures 3C, D**). Suppression of these pathways favors IEC differentiation into specific cell lineages, such as absorptive and secretory cell types.

### IL-22 strengthens innate immune and barrier signaling in infant enteroids

**Figure 4:**
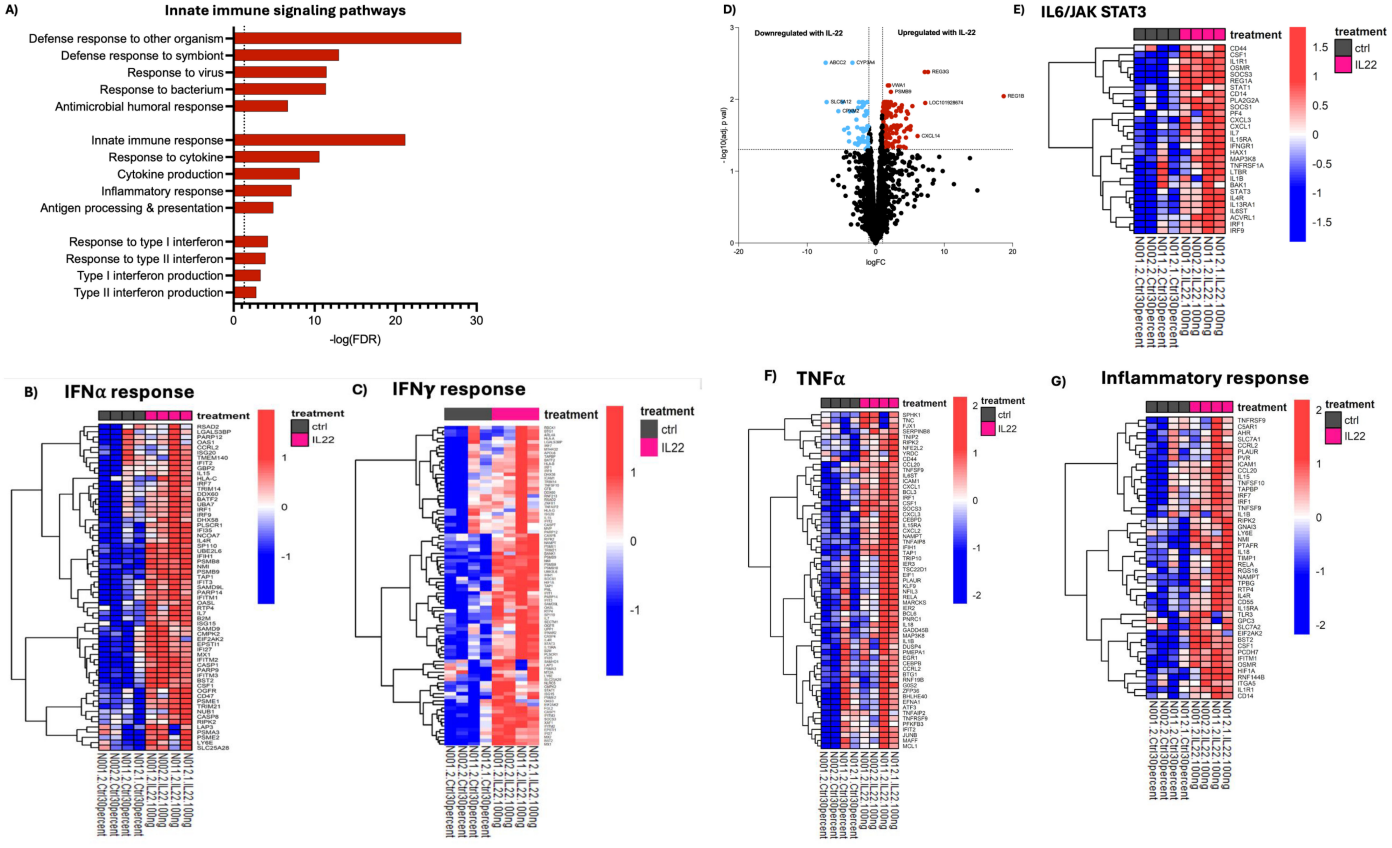
IL-22 induces genes involved in innate immune functions in developing enteroids. Gene set enrichment analysis was performed comparing IL-22 treated to untreated enteroids. **(A)** Enrichment of selected GO pathways related to innate immune response signaling is shown. **(B, C, E–G)** Depict leading-edge genes for indicated pathways from GSEA using the Hallmark database. Color represents Z-scores. **(D)** Infant enteroids treated with IL-22 displayed upregulation of genes encoding antimicrobial peptides as indicated by the volcano plot of fold-changes in gene expression vs FDR-corrected p values. Blue downregulated, red-upregulated. Enteroid samples used N001, N002, N011 and N012.

During development, IECs are central in maintaining gut homeostasis, mediated in part through their interactions with, and responses to signals from immune cells via receptor binding, signal transduction, and subsequent transcriptional activation (1, 2, 35, 37). These processes have largely been studied in the adult intestine and are comparatively poorly defined in the infant GI tract. We sought to examine innate immune and barrier-protective pathways in infant enteroids using our rich transcriptomic data to elucidate IL-22’s role in this context. Following treatment with IL-22, a notable increase in innate immune signaling pathways (*REG3G, UBE2L6, DTX3L, HK1, CD55, ANXA1, IRF7, PLSCR1, IRF1, CFB*) was observed (**Figure 4A**). For example, we noted significant enrichment in genes involved in defense against other organisms (*REG3A, REG3G, REG1B*) including bacteria symbionts (*DTX3L, IRF7, PLSCR1, IRF1, IFIH1*) and genes that regulate cellular responses to cytokines (*IL7, SBNO2, IL15RA, IRF7* and *PARP9*). IL-22 treatment was also accompanied by an increase in interferon (IFN)-α and -γ response pathways (**Figures 4B, C**). Secretion of antimicrobial factors by the intestinal epithelium mediates a careful balance between host and its enormous population of resident luminal gut microbes. As noted earlier, transcription of the genes encoding REG3α and REG1ß were markedly upregulated in IL-22-treated enteroids (**Figure 4D**). These antimicrobial peptides are released by Paneth cells in the small intestine, helping to protect the host from enteric bacterial infections. These findings mimic observations of early life gut development, where an important role for antimicrobial peptides and innate immune signaling has been reported (14). Among other affected pathways, we found that IL-22 strongly induced genes involved in the regulation of IL6-JAK-STAT signaling (**Figure 4E**), a known downstream target of IL-22, and critical in coordinating inflammation and cellular metabolism under homeostatic and pathological conditions (38). We also found upregulation of genes associated with TNF-α signaling and inflammatory response pathways in IL-22-treated enteroids (**Figures 4F, G**). Under healthy/homeostatic conditions, these pathways contribute to immune surveillance, including activation of immune cells, tissue repair, and homeostasis. In contrast, they can also promote inflammation when dysregulated, such as seen in patients with Crohn’s disease or ulcerative colitis (39). Our data also revealed marked changes in the adaptive immune response, including enrichment of genes associated with T-cell mediated responses in our IL-22 treated enteroids (**Supplementary Figure 2** **).** Overall, our data supports a potentially beneficial role for IL-22 signaling in early-life gut protection and maturation.

## Discussion

Infants can prove exceptionally vulnerable to intestinal insults during their first year of life, which can have devastating and even fatal consequences. How the intestinal epithelium develops in infancy and protects itself from noxious luminal stimuli is still poorly defined (14–16). In this study, we developed an *in vitro* gut organoid model to study developmental-specific gut function in human infants. We propose that the gut epithelium’s function of those infants largely depends on intricate crosstalk between IECs and nearby immune cells within the intestinal mucosa. For ethical reasons, our understanding of these processes, particularly in healthy infants, remains poorly defined. Yet, with the increasing incidence of severe GI conditions among children (10–12), there is a pressing need to establish non-invasive models to investigate normal gut development, as well as perturbed intestinal maturation in early life, that can have life-long health consequences, such as NEC and IBD. Using this approach, we demonstrated that enteroid models derived from ileal tissues collected from infants under six months of age likely recapitulate the early-life intestinal microenvironment, fostering intestinal epithelial growth and development.

In a previous study, we determined that neonatal Th17 cells show a developmentally altered cytokine profile that, unlike adult Th17 cells, is skewed towards IL-22, with only modest production of IL-17A (29). To our knowledge, the stage of human development at which Th17 cells switch from IL-22 to preferential IL-17 production has not been clearly defined in the literature. We also showed that this age-dependent cytokine profile was influenced by TGF-β in cord blood (29). Finding that neutralization of IL-22 within the infant Th17-conditioned media abrogated its effects on infant enteroid responses prompted us to further examine the physiological effect of IL-22 on the development and function of infant IECs. In this study, we found that IL-22-treated enteroids exhibited enhanced growth and proliferation, marked by increased size and other morphological changes, as well as concurrent increases in expression of the proliferation marker Ki67. These phenotypes were ablated in enteroids exposed to neonatal Th17 cells-derived conditioned media when neutralizing IL-22 was also added, confirming the effects linked to this cytokine.

Our data revealed increased expression of several antimicrobial genes, including REG3G and REG1B. These findings are consistent with work from Pavildis et al., who found significant increases in antimicrobial peptide genes following IL-22 treatment of colonoids (40). It should be noted, however, that these genes are not only restricted to Paneth cells but also expressed by IECs following stimulation by IL-22 (45). Aside from IL-22, neonatal Th17 cells also produce IL-10, which is an important cytokine known for its anti-inflammatory role in the gut (41). We did not, examine IL-10 in our study, but plan to do so in future studies (24).

Both animal and human models have confirmed a proliferative and pro-regenerative role for IL-22 acting on different epithelial subsets (42), including TA and Paneth cells (25, 26, 43), as well as stimulating the release of mucus and AMP production (43). Transcriptomic profiling of our enteroids identified significantly more IEC subtypes in IL-22 treated samples than in controls, with upregulated signatures for TA, progenitor, stem, and Paneth cells. We also noted a trend towards more goblet cell signature, although it did not reach the significance seen with immunofluorescence staining for MUC2. This may reflect differences in the degree of goblet cell differentiation amongst cultures, or differential timing for protein versus mRNA markers. These findings suggest that crosstalk between IECs and IL-22 may underlie, in part, the functional maturation of the infant intestinal epithelium, with the differentiation into various IEC subtypes promoting gut development, innate immune function, and barrier protection in early life. RNA-seq analysis also revealed that IL-22 reduces expression of the Wnt and NOTCH pathways, which favors differentiation, particularly of secretory cell lineages.

The IL-6-JAK-STAT pathway regulates IEC proliferation, differentiation, and survival, all of which are critical for maintaining the integrity and function of the intestinal barrier (44). IL-6 activation of this pathway leads to the phosphorylation of JAKs, followed by the activation of STAT proteins. This pathway also plays an essential role in promoting local inflammation and immune responses in the gut as well as systemically. Here, we found evidence that IL-22 significantly upregulated the IL-6-JAK-STAT pathways in our enteroids. We hypothesize that, in a healthy gut, activation of the IL-6-JAK-STAT pathway helps promote a heightened state of immune readiness in IECs (45). Such functions could play a pivotal role in ensuring that the infant’s GI tract rapidly responds to luminal threats. Moreover, the upregulation of genes associated with interferon and TNF-α signaling suggests a type of innate immune readiness and inflammatory tone essential for the developing gut epithelium. This may serve as a protective strategy for the infant’s gut while the immune system fully matures (45).

There are many ways in which infant enteroids can be used to advance research that could benefit this age group specifically, including, for example, examining the impact of early life nutrition, such as breast milk (i.e., human milk oligosaccharides) versus formula on IEC development and function. Infection of these enteroids by different diarrheal pathogens, such as rotavirus, could help refine mechanisms involved in early-life enteric infections and diarrhea, which are common causes of mortality in infants across the world. Combining the model with other high throughput methods could help further explore responses to certain drugs, probiotics, or nutrients for their impact on gut health, further advancing personalized medicine in infants.

In summary, using a human infant enteroid model, we demonstrated the important role of IL-22 on the developing epithelium, both in terms of morphological and functional changes in IECs. Our findings suggest that IL-22 likely mediates crosstalk between IECs and nearby Th17 cells in the infant GI tract to support the growth, maturation, and protection of the developing infant gut.

## Data Availability

Gene expression data have been submitted to the NCBI Gene Expression Omnibus repository under the accession number GSE282290.

## References

[1] de Santa Barbara P van den Brink GR Roberts DJ. Development and differentiation of the intestinal epithelium. Cell Mol Life Sci. (2003) 60:1322–32. doi: 10.1007/s00018-003-2289-3 PMC2435618 12943221

[2] Frazer LC Good M. Intestinal epithelium in early life. Mucosal Immunol. (2022) 15:1181–7. doi: 10.1038/s41385-022-00579-8 PMC10329854 36380094

[3] Kalbermatter C Fernandez Trigo N Christensen S Ganal-Vonarburg SC. Maternal microbiota, early life colonization and breast milk drive immune development in the newborn. Front Immunol. (2021) 12:683022. doi: 10.3389/fimmu.2021.683022 34054875 PMC8158941

[4] Torow N Marsland BJ Hornef MW Gollwitzer ES. Neonatal mucosal immunology. Mucosal Immunol. (2017) 10:5–17. doi: 10.1038/mi.2016.81 27649929

[5] Neu J Walker WA. Necrotizing enterocolitis. N Engl J Med. (2011) 364:255–64. doi: 10.1056/NEJMra1005408 PMC3628622 21247316

[6] Stenson WF. Postnatal growth in the intestine. Curr Opin Gastroenterol. (2013) 29:107–11. doi: 10.1097/MOG.0b013e32835d9ec3 23286926

[7] Pelaseyed T Bergström JH Gustafsson JK Ermund A Birchenough GMH Schütte A. The mucus and mucins of the goblet cells and enterocytes provide the first defense line of the gastrointestinal tract and interact with the immune system. Immunol Rev. (2014) 260(1):8–20. doi: 10.1111/imr.12182 24942678 PMC4281373

[8] Garcia AM Fadel SA Cao S Sarzotti M. T cell immunity in neonates. Immunol Res. (2000) 22:177–90. doi: 10.1385/IR:22:2-3:177 11339354

[9] Egan CE Sodhi CP Good M Lin J Jia H Yamaguchi Y. Toll-like receptor 4–mediated lymphocyte influx induces neonatal necrotizing enterocolitis. J Clin Investigation. (2015) 126(2):495. doi: 10.1172/JCI83356 PMC4731173 26690704

[10] Long D Wang C Huang Y Mao C Xu Y Zhu Y. Changing epidemiology of inflammatory bowel disease in children and adolescents. Int J Colorectal Dis. (2024) 39:73. doi: 10.1007/s00384-024-04640-9 38760622 PMC11101569

[11] Ibrahim ATA Hamdy AM Elhodhod MA. Prevalence of Functional Gastrointestinal Disorders among School-aged Children and adolescents, A Multicenter Study. QJM: Int J Medicine. (2020) 113:hcaa063.029. doi: 10.1093/qjmed/hcaa063.029

[12] Kuenzig ME Fung SG Marderfeld L Mak JWY Kaplan GG Ng SC. Twenty-first century trends in the global epidemiology of pediatric-onset inflammatory bowel disease: systematic review. Gastroenterology. (2022) 162(4):1147–59.e4. doi: 10.1053/j.gastro.2021.12.282 34995526

[13] Vicente AM Ballensiefen W Jönsson JI. How personalised medicine will transform healthcare by 2030: the ICPerMed vision. J Trans Medicine. (2020) 18:180. doi: 10.1186/s12967-020-02316-w PMC7189458 32345312

[14] Georgountzou A Papadopoulos NG. Postnatal innate immune development: from birth to adulthood. Front Immunol. (2017) 8:957. doi: 10.3389/fimmu.2017.00957 28848557 PMC5554489

[15] Pandey U Aich P. Postnatal intestinal mucosa and gut microbial composition develop hand in hand: A mouse study. Biomed J. (2022) 46:100519. doi: 10.1016/j.bj.2022.03.004 35306225 PMC10267966

[16] Basha S Surendran N Pichichero M. Immune responses in neonates. Expert Rev Clin Immunol. (2014) 10:1171. doi: 10.1586/1744666X.2014.942288 25088080 PMC4407563

[17] Meran L Baulies A Li VSW. Intestinal stem cell niche: the extracellular matrix and cellular components. Stem Cells Int. (2017) 2017:7970385. doi: 10.1155/2017/7970385 28835755 PMC5556610

[18] Angus HCK Butt AG Schultz M Kemp RA. Intestinal organoids as a tool for inflammatory bowel disease research. Front Med (Lausanne). (2020) 6:334. doi: 10.3389/fmed.2019.00334 32010704 PMC6978713

[19] Kretzschmar K Clevers H. Organoids: modeling development and the stem cell niche in a dish. Dev Cell. (2016) 38:590–600. doi: 10.1016/j.devcel.2016.08.014 27676432

[20] Co JY Klein JA Kang S Homan KA. Suspended hydrogel culture as a method to scale up intestinal organoids. Sci Rep. (2023) 13:10412. doi: 10.1038/s41598-023-35657-9 37369732 PMC10300005

[21] Pleguezuelos-Manzano C Puschhof J van den Brink S Geurts V Beumer J Clevers H. Establishment and culture of human intestinal organoids derived from adult stem cells. Curr Protoc Immunol. (2020) 130:e106. doi: 10.1002/cpim.106 32940424 PMC9285512

[22] Crowley SM Han X Allaire JM Stahl M Rauch I Knodler LA. Intestinal restriction of Salmonella Typhimurium requires caspase-1 and caspase-11 epithelial intrinsic inflammasomes. PLoS Pathogens. (2020) 16(4):e1008498. doi: 10.1371/journal.ppat.1008498 32282854 PMC7179941

[23] Cook L Stahl M Han X Nazli A MacDonald KN Wong MQ. Suppressive and gut-reparative functions of human type 1 T regulatory cells. Gastroenterology. (2019) 157(6):1584–98. doi: 10.1053/j.gastro.2019.09.002 31513797

[24] He GW Lin L DeMartino J Zheng X Staliarova N Dayton T. Optimized human intestinal organoid model reveals interleukin-22-dependency of paneth cell formation. Cell Stem Cell. (2022) 29(9):1333–45.e6. doi: 10.1016/j.stem.2022.08.002 36002022 PMC9438971

[25] Zha JM Li HS Lin Q Kuo WT Jiang ZH Tsai PY. Interleukin 22 expands transit-amplifying cells while depleting lgr5+ Stem cells via inhibition of wnt and notch signaling. Cell Mol Gastroenterol Hepatol. (2019) 7(2):255–74. doi: 10.1016/j.jcmgh.2018.09.006 PMC6352747 30686779

[26] Zwarycz B Gracz AD Rivera KR Williamson IA Samsa LA Starmer J. IL22 inhibits epithelial stem cell expansion in an ileal organoid model. Cell Mol Gastroenterol Hepatol. (2019) 7(1):1–17. doi: 10.1016/j.jcmgh.2018.06.008 30364840 PMC6199238

[27] Gritz EC Bhandari V. The human neonatal gut microbiome: A brief review. Front Pediatr. (2015) 3:17. doi: 10.3389/fped.2015.00017 25798435 PMC4350424

[28] Tsafaras GP Ntontsi P Xanthou G. Advantages and limitations of the neonatal immune system. Front Pediatr. (2020) 8:5. doi: 10.3389/fped.2020.00005 32047730 PMC6997472

[29] Razzaghian HR Sharafian Z Sharma AA Boyce GK Lee K Da Silva R. Neonatal T helper 17 responses are skewed towards an immunoregulatory interleukin-22 phenotype. Front Immunol. (2021) 12:655027. doi: 10.3389/fimmu.2021.655027 34012439 PMC8126652

[30] Burgler S Ouaked N Bassin C Basinski TM Mantel PY Siegmund K. Differentiation and functional analysis of human T(H)17 cells. J Allergy Clin Immunol. (2009) 123(3):588–595, 595.e1-7. doi: 10.1016/j.jaci.2008.12.017 19178935

[31] Allaire JM Poon A Crowley SM Han X Sharafian Z Moore N. Interleukin-37 regulates innate immune signaling in human and mouse colonic organoids. Sci Rep. (2021) 11(1):8206. doi: 10.1038/s41598-021-87592-2 33859245 PMC8050237

[32] Gundem G Lopez-Bigas N. Sample-level enrichment analysis unravels shared stress phenotypes among multiple cancer types. Genome Med. (2012) 4:28. doi: 10.1186/gm327 22458606 PMC3446278

[33] Wang Y Song W Wang J Wang T Xiong X Qi Z. Single-cell transcriptome analysis reveals differential nutrient absorption functions in human intestine. J Exp Med. (2020) 217(2):e20191130. doi: 10.1084/jem.20191130 31753849 PMC7041720

[34] Lavoie PM Huang Q Jolette E Whalen M Nuyt AM Audibert F. Profound lack of interleukin (IL)-12/IL-23p40 in neonates born early in gestation is associated with an increased risk of sepsis. J Infect Diseases. (2010) 202(11):1754. doi: 10.1086/657143 20977341 PMC2975517

[35] Kolev HM Kaestner KH. Mammalian intestinal development and differentiation—The state of the art. Cell Mol Gastroenterol Hepatol. (2023) 16:809–21. doi: 10.1016/j.jcmgh.2023.07.011 PMC10520362 37507088

[36] Cancedda R Mastrogiacomo M. Transit Amplifying Cells (TACs): a still not fully understood cell population. Front Bioeng Biotechnol. (2023) 11:1189225. doi: 10.3389/fbioe.2023.1189225 37229487 PMC10203484

[37] Beumer J Clevers H. Regulation and plasticity of intestinal stem cells during homeostasis and regeneration. Development. (2016) 143:3639–49. doi: 10.1242/dev.133132 27802133

[38] Lejeune D Dumoutier L Constantinescu S Kruijer W Schuringa JJ Renauld JC. Interleukin-22 (IL-22) Activates the JAK/STAT, ERK, JNK, and p38 MAP Kinase Pathways in a Rat Hepatoma Cell Line: pathways that are shared with and distinct from IL-10*. J Biol Chem. (2002) 277:33676–82. doi: 10.1074/jbc.M204204200 12087100

[39] Pagnini C Cominelli F. Tumor necrosis factor’s pathway in crohn’s disease: potential for intervention. Int J Mol Sci. (2021) 22:10273. doi: 10.3390/ijms221910273 34638616 PMC8508644

[40] Pavlidis P Tsakmaki A Pantazi E Li K Cozzetto D Digby- Bell J. Interleukin-22 regulates neutrophil recruitment in ulcerative colitis and is associated with resistance to ustekinumab therapy. Nat Commun. (2022) 13(1):5820. doi: 10.1038/s41467-022-33331-8 36192482 PMC9530232

[41] Nguyen HD Aljamaei HM Stadnyk AW. The production and function of endogenous interleukin-10 in intestinal epithelial cells and gut homeostasis. Cell Mol Gastroenterol Hepatol. (2021) 12:1343–52. doi: 10.1016/j.jcmgh.2021.07.005 PMC8463866 34271223

[42] Lindemans CA Calafiore M Mertelsmann AM O’Connor MH Dudakov JA Jenq RR. Interleukin-22 promotes intestinal-stem-cell-mediated epithelial regeneration. Nature. (2015) 528(7583):560–4. doi: 10.1038/nature16460 PMC4720437 26649819

[43] Patnaude L Mayo M Mario R Wu X Knight H Creamer K. Mechanisms and regulation of IL-22-mediated intestinal epithelial homeostasis and repair. Life Sci. (2021) 271:119195. doi: 10.1016/j.lfs.2021.119195 33581125

[44] Salas A Hernandez-Rocha C Duijvestein M Faubion W McGovern D Vermeire S. JAK–STAT pathway targeting for the treatment of inflammatory bowel disease. Nat Rev Gastroenterol Hepatol. (2020) 17(6):323–37. doi: 10.1038/s41575-020-0273-0 32203403

[45] Miyake K Fukui R. Chapter 2 - homeostatic inflammation as environmental-adaptation strategy. In: Amadori M, editor. The Innate Immune Response to Noninfectious Stressors [Internet]. Academic Press (2016). p. 25–52. doi: 10.1016/B978-0-12-801968-9.00002-7

