# Supplementary material for: Crosstalk with infant-derived Th17 cells, as well as exposure to IL-22 promotes maturation of intestinal epithelial cells in an enteroid model
Source: Front Immunol. 2025 May 1;16:1582688. doi: 10.3389/fimmu.2025.1582688 (PMC12078324; doi:10.3389/fimmu.2025.1582688)
Supplement: Supplementary file 1 [file DataSheet1.pdf]

Supplemental Materials

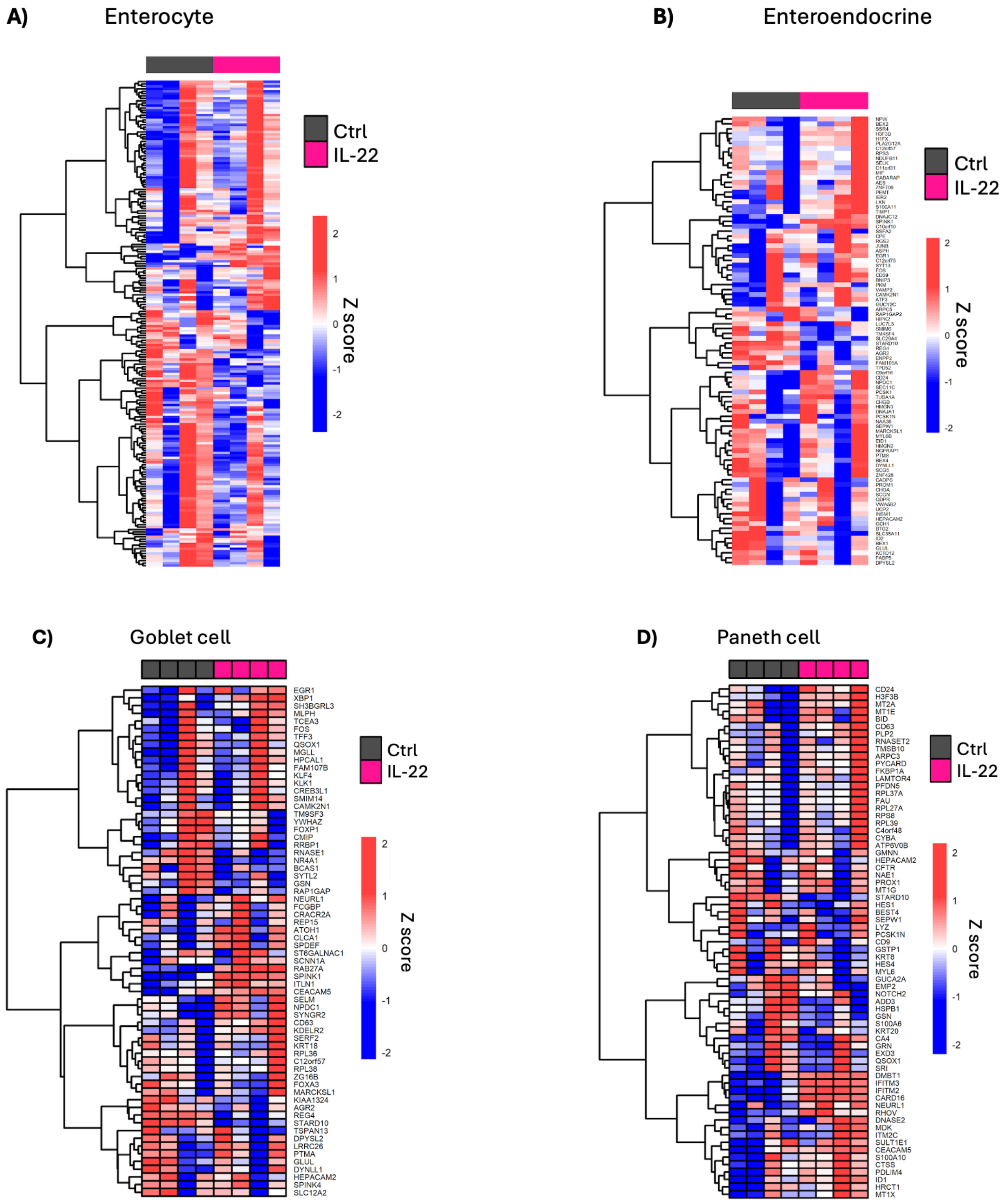

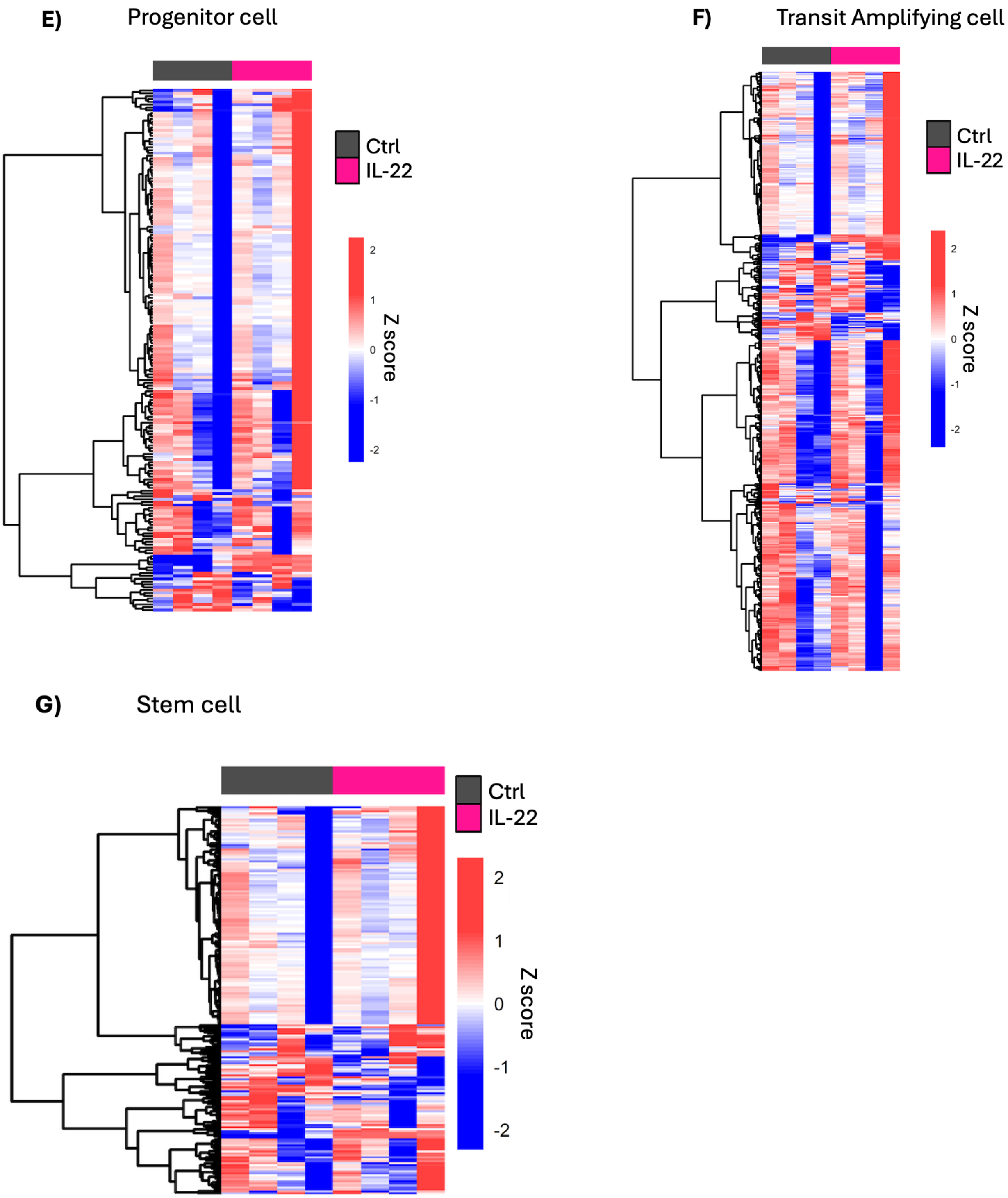

Supplemental Figure 1: Transcriptomic identification of cell types in infant enteroids. A) Enterocyte, B) Enteroendocrine, C) Goblet, D) Paneth, E) Progenitor, F) Transit amplifying and G) Stem cells. Enteroid samples used N001, N002, N011 and N012.

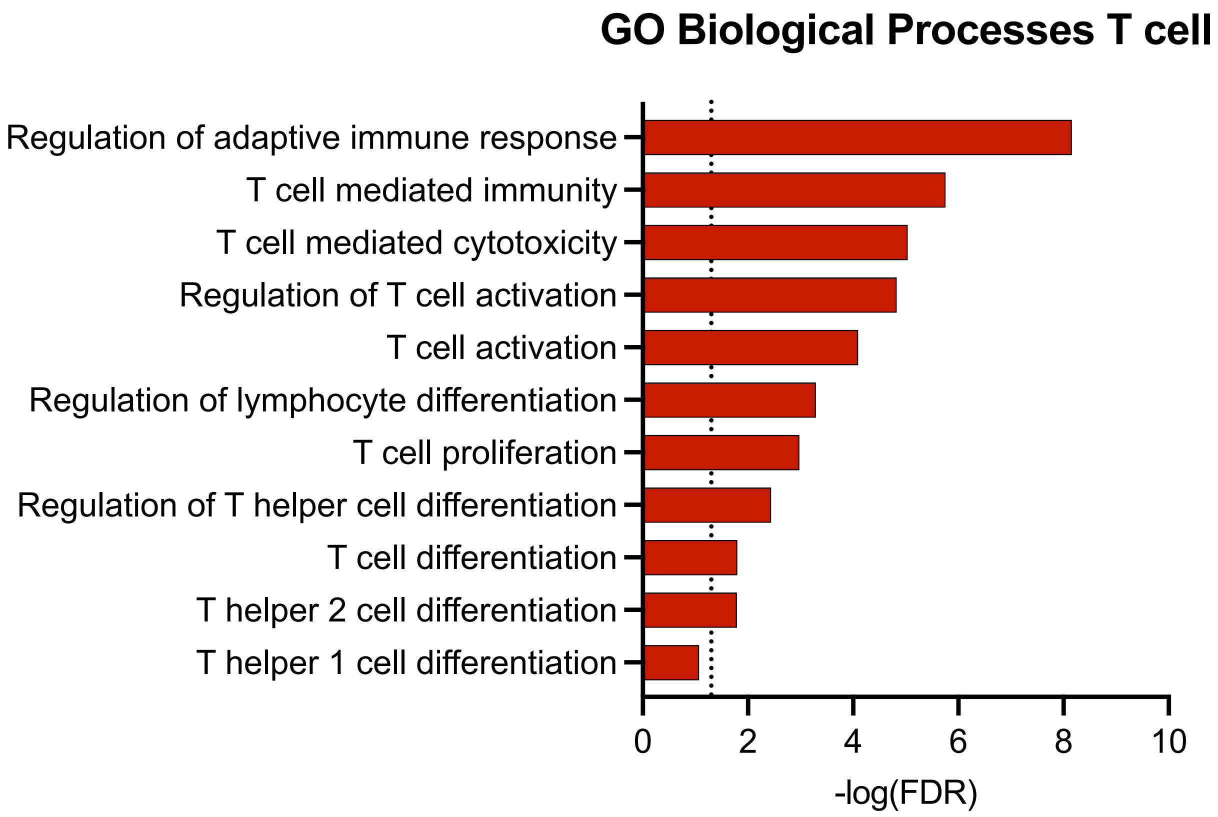

Supplemental Figure 2: Transcriptomic profile of T-cell mediated response in infant enteroids in response to IL-22.

Protocol for generation and stimulation of infant enteroids

Generation of enteroids

Enteroids were generated from terminal ileum tissue using a previously established protocol.[43]

1. Wash biopsy samples 4-5 times in 5 mL 1X cold chelating solution (CCS) until clear. Once clear, resuspend samples in 10 mL of cold expanded CCS.
2. Incubate biopsies at 4°C on a shaker for 45 minutes (600 strokes/minute).
3. Remove biopsy samples and finely chopped using a straight edge razor blade and resuspend in cold MEM+++.
4. Spin samples at 1200rpm for 5 minutes at 4°C. Remove supernatant and add 500 µL of cold MEM+++.
5. Spin at 1500 rpm for 5 minutes at 4°C.
6. After washing/spinning, remove supernatant and resuspend pellet in cold MEM+++ according to the appropriate calculated volume. Add cold Matrigel (Corning) 1:1 at (40 µL/well) to the sample/MEM+++ mixture and mix thoroughly to homogenize. Be careful not to allow the Matrigel to warm or it will solidify in the tube.
7. Using a p200 pipette, add Matrigel domes of 40 µL in the center of wells in a prewarmed 24-well culture plate (Corning).
8. Incubate plates at 37°C for 15-30 minutes to ensure solidification of Matrigel.
9. Add 500 µL of organoid growth medium containing media and incubate at 37°C, 5% CO_2.
10. Change culture media every two days (400 µL/well).

11. Passage enteroids every 7 to 10 days, depending on growth rate and size needed for further experiments. Enteroids utilized in this experiment were developed for 3-5 passages.

Reagents and buffers

Reagent	Concentration/percent	Volume for 50 ml
MEM+++	25 ml Advanced DMEM/F12 supplemented with 1% Penicillin/Streptomycin, 1% Glutamax, and 1M HEPES	25 mL
Chelating solution	1X	5 mL
Chelating solution expanded	Supplement with 200 µL 0.5 M EDTA and 100 µL of Gentamicin	

Organoid growth medium:

Reagent	Concentration/percent	Volume for 50 ml
MEM+++ 25ml Advanced DMEM/F12	Supplement with 1% Penicillin/Streptomycin, 1% Glutamax, and 1M HEPES	25 mL
WRN (Wnt3a, Rspondin-3, and Noggin) conditioning medium		25 mL
B27 supplement (Invitrogen)	1000 µL	
N2 supplement (Invitrogen)	500 µL	
N-acetylcysteine (Sigma-Aldrich)	125 µL (500mM stock)	
Nicotinamide (Sigma-Aldrich)	500 µL (1M, stock)	
mEGF (Invitrogen)	50 µL (1000X)	
A83-01 supplement TGF-B inhibitor (Tocris)	50 µL (1000x stock)	
Rock inhibitor (Y27632) (AbMole)	50 µL (1000x stock)	
SB202190 supplement P38 inhibitor (Sigma-Aldrich)	16.5 µL (3000x stock)	
*Filter Sterilize (0.2um filter)		

Naïve CD4 T cell isolation, differentiation and measurement of IL-22

1. Cord blood was collected from term deliveries by Cesarean section (>38-41 weeks of gestation) in sodium heparin anti-coagulated vacutainers (Becton Dickinson, Canada), and processed within 2-6 hours of collection.
2. Cord blood mononuclear cells (CBMCs) were isolated from de-identified cord blood samples via density centrifugation with LymphoPrep (STEMCELL Technologies), cryopreserved in

PBS with 44 mg/mL human serum albumin, 2mM EDTA, and 10% DMSO (1.0 x 106 cells/mL), and stored at -80°C.

3. Naïve CD4+ T cells were isolated from thawed CBMCs via immunomagnetic negative selection (StemCell Kit), manually counted, and seeded in a 96-well round-bottom plate (0.5 x 106 cells per well). As previously described,[25] activation of CD4 T cells was achieved using anti-CD3 and anti- CD28 soluble antibody complexes (25 µL/mL; ImmunoCult™ Human CD3/CD28 T Cell Activator) in complete RPMI (RPMI-1640 medium supplemented with 10 % human AB serum, 1 % sodium pyruvate, 1 % 1X Penicillin/Streptomycin) in the presence of human recombinant IL-6 (20 ng/mL), IL-23 (10 ng/mL), IL-1β (10 ng/mL) (Peprotech) and neutralizing IL-4 and IFN-γ antibodies (RATIO; BD Pharmingen) at 37°C (5% CO_2) for seven days for differentiation of näive CD4+ T cells to Th17 cells.
4. Fresh media and cytokines were added after 3 days.
5. Supernatants were collected at the end of the culture and frozen at -80°C in batches.
6. IL-22 was measured in adult (blue) and neonatal (pink) magnetic bead-purified naïve CD4 T cells activated by anti-CD3/CD38 soluble antibody complex for 7 days in the presence of recombinant IL-6 (20 ng/mL), IL-23 (10ng/mL), IL-1β (10 ng/mL), and neutralizing IL-4 and IFN-γ antibodies. IL-22 concentration in culture supernatants was determined by ELISA (Biolegend IL-22 Kit).

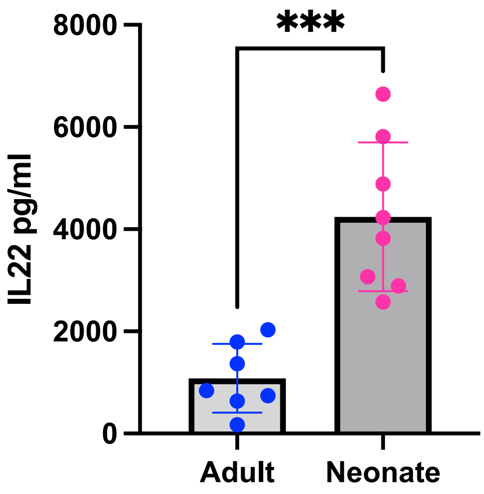

Level of IL-22 concentration in adult and neonatal activated CD4 T-cells.

Stimulation of human enteroids

1. Enteroids were passaged as described above (Step 9).
2. After culturing for 4 days, organoid media was replaced with fresh 70% organoid growth medium supplemented either with neonatalTh17 culture supernatant or cRPMI as a control condition.
3. For conditions requiring recombinant cytokines, IL-22 was added to the growth organoids medium with 30 % of cRPMI. Recombinant Il-22 (R&D Systems) was used at 100 ng/ml. Neutralizing Il-22 (R&D Systems) was also used at 5 µg/ml.
4. On Day 7, enteroid cultures were collected, pooled, and harvested for further analysis.

Sample information

ID	Sex	Gestational age at birth (weeks)	Postnatal age (weeks)	Location of biopsy	Organoid growth
N001	F	32	9	Ileum	Good
N002	M	33	12	Ileum	Good
N005	F	38	10	Ileum	Good
N006	F	33	6	Ileum	Inconsistent or weak
N009	F	26	11	Ileum	Inconsistent or weak
N010	F	40	9	Ileum	Good
N011	M	33	19	Ileum	Good
N012	F	37	7.5	Ileum	Good
N013	M	36	12	Ileum	Not recovered
N014	M	29	10	Ileum	Slow recovering
